# Human-wildlife conflict and community perceptions towards wildlife conservation in and around Wof-Washa Natural State Forest, Ethiopia

**DOI:** 10.1186/s40850-022-00154-5

**Published:** 2022-09-27

**Authors:** Dereje Yazezew

**Affiliations:** Department of Biology, College of Natural and Computational Science, Debre Berhan University, P. O. Box 445, Debre Berhan, Ethiopia

**Keywords:** Biodiversity conservation, Crop damage, Grivet monkey, Local communities, Respondents perceptions

## Abstract

**Background:**

Human-wildlife conflict (HWC) is forecasted to increase globally in the vicinity of protected areas and covers various dimensions. It occurs in several different contexts and involves a range of animal taxonomic groups where the needs and requirements intersect with humans’ needs and development. More often, human-monkey conflict occurs in developing countries and is amongst the main threats to biodiversity conservation in these regions. Grivet monkeys are slender agile monkeys of the genus *Cercopithecus.* This study was conducted to investigate the status of human grivet monkey conflict and the attitude of local communities towards grivet monkey conservation in and around Wof-Washa Natural State Forest (WWNSF), Ethiopia from September 2017 to May 2018. A questionnaire survey (143 respondents) was used to study the human-grivet monkey conflict and its conservation status.

**Results:**

The majority of respondents (male = 67.1%; female = 74.1%) were not supporting grivet monkey conservation due to the troublesome crop-damaging effect of the animal. Respondents having settlements/farmland nearer to the forest have significantly negative perceptions towards grivet monkey conservation than those far from it. The majority of respondents opined that eradication/relocation of grivet monkeys and financial compensation are the options to mitigate human-grivet monkey conflict. Based on the questionnaire result, 42.5 ± SD 8.68 of respondents in all villages elucidated that the main cause of crop damage by grivet monkeys was habitat degradation.

**Conclusion:**

In the study area, Human-Grivet Monkey Conflict (HGMC) is exacerbated by the encroachment of local communities into the forest area, exploitation of resources that would be used by grivet monkeys, and enhanced crop damage by grivet monkeys. As a result grivet monkeys have been persecuted as a consequence of crop damage. This was due to the negative attitude developed from human perspective. Thus, awareness creation education programs and feasible crop damage prevention techniques need to be implemented.

**Supplementary Information:**

The online version contains supplementary material available at 10.1186/s40850-022-00154-5.

## Introduction

Human-wildlife conflict (HWC) is predicted to increase globally and occurs in several different contexts and spans a range of animal taxonomic groups [1, 2]. Currently, HWC is a global issue that has adverse consequences for both humans and wildlife [3]. Human-Wildlife Conflict (HWC) arises from a range of direct and indirect negative interactions between humans and wildlife. This occurs when the needs and requirements of humans and wildlife overlap, which usually results in costs to both the local residents and animals when the needs of one impact negatively on the other [1, 4]. The loss, degradation, and fragmentation of habitats through human activities such as logging, animal husbandry, hunting, agricultural expansion and developmental projects and other such factors [5–7] intensify the conflict and affected primate populations [8]. Ecosystems and habitats are alarmingly dominated by humans, which trigger species, including primates, to exploit new human resources to survive [9, 10].

Accordingly, primates and humans are often in potential conflict over crops due to primates’ renowned crop foraging behavior. Among wild animal species that cause damage to farmers’ yield and trigger HWC, primates take the top ranking [11]. The genera *Cercopithicus, Papio* and *Macaca*, particularly baboons and grivet monkeys are some of the most serious crop foragers because of their intelligence, adaptability, wide dietary range, complex social organization, and aggression [6]. As human populations expand and natural habitats shrink, people and animals are increasingly coming into conflict over living space and food. Although feeding on cereal crops increases foraging efficiency and nutrient intake for primates, it is nuisance for farmers due to crop loss [12, 13].

Grivet monkeys *(C. aethiops)* are slender agile monkeys of the genus *Cercopithecus*, inhabiting wooded regions of Africa and are the most widely distributed of the guenon species. They occur from Ethiopia to Senegal and from Sudan to South Africa [14]. So far, this species is considered to be widely distributed and often found in northern and central Ethiopia at altitudes ranging from near sea level to approximately 3000 m a.s.l. [15]. However, because of habitat fragmentation by human settlement and cultivation into previous wildlife habitats, the distribution of grivet monkeys is adversely affected nowadays. In many areas, this monkey frequents human settlements and feeds extensively on crops [15–17], which exacerbates the conflict with humans.

One of the important issues in wildlife conservation is managing human-wildlife conflicts in habitats dominated by humans. Understanding past and present patterns of conflict or species distribution directs the way and paves the road for sustainable livelihood improvement of communities and wildlife conservation. Thus, the study of human-grivet monkeys conflict in the study area is crucial to designing a feasible and resilient conservation plan for the study species and other animals inhabiting in Wof-Washa Natural State Forest. Accordingly, longitudinal studies on conservation threats of primates are an important step toward developing effective conservation management plans. In spite of this governing rationale, only a few studies have been conducted in the central highlands of Ethiopia where the conflict is severe due to a high rate of forest degradation and restriction of primates to patches of habitat surrounded by agricultural fields [16]. This study aimed to provide baseline information on the current situation of human-grivet monkey conflict (HGMC) and the perceptions of communities towards this monkey conservation. The researcher predicted that HGMC did not vary with regard to the distance of farmland from the forest boundary. The researcher also predicted that the attitude of households may not vary depending on gender.

## Results

### Demographic characteristics and socioeconomic profile of the respondents

A total of 143 individuals participated in the questionnaire survey (Table 1). The majority of respondents 59.4% (*n* = 85) were males, while 40.6% (*n* = 58) were females. There was a significant difference in the number of male and female respondents participated in the interview (χ2 = 5.10, df = 1, *P* = 0.024). Most of the respondents 81.1% (*n* = 116) were married. Regarding educational level, 83.9% (*n* = 120) were literate, and 16.1% (*n* = 23) were uneducated. The family sizes of the respondents ranged from 1 to 11 with a mean of 4.82 ± 2.13. Of the total respondents, 52.4% (*n* = 75) possess a family size of 4 to 6 individuals. There was a significant difference in family size among villages (χ2 = 21.82, df = 2, *P* = 0.016). Among the households, 53.1% (*n* = 75) possess 0.5-1 ha of farmland while few 17.5% (n = 25) possess > 1 ha of farmland. There was a significant difference among villages regarding farmland size (χ2 = 19.52, df = 2, *P* = 0.034). The majority of respondents 84.6% (*n* = 121) lack private and communal land and leave their cattle to graze in the forest while only 11.9% (*n* = 17) possess private grazing land. There was a significant difference in the proportion of households owning grazing land (T test: *t* = 59.6, df = 2, *P* = 0.000). The livelihoods of respondents were subsistence farming where they reared livestock and cultivated different crops like barley (*Hordeum vulgare L.*), wheat (*Triticum aestivum L.*), bean (*Vicia faba L.*), maize (*Zea mays*), pea (*Pisum sativum L.*), and lentil (*Lens culinaris Medikus*).

**Table 1:** Demographic characteristics and socioeconomic profile of respondents around Wof-Washa Natural State Forest (WWNSF)

characteristics	Category	N (frequency)	% (percent)
Age	18−30	41	28.7
	31−43	48	33.6
	44−56	31	21.7
	> 57	23	16.1
Sex	Male	85	59.4
	Female	58	40.6
Marital Status	Married	116	81.1
	Single	27	18.9
Education	Uneducated	23	16.1
	informal education	48	33.6
	primary	50	35.0
	secondary	22	15.4
Family size	1−3	36	25.2
	4 − 6	75	52.4
	> 7	32	22.4
Source of livelihood	Crop cultivation	5	3.5
	Crop & livestock	138	96.5
Farmland size	< 0.5 ha	42	29.4
	0.5−1 ha	76	53.1
	> 1 ha	25	17.5
Number of livestock	0−5	25	17.5
	5−10	78	54.5
	15−Nov	36	25.2
	> 15	4	2.8
Grazing land	No, in barn	5	3.5
	Yes, private	17	11.9
	No, in the forest	121	84.6

### Human-grivet monkey conflict

Based on the questionnaire survey, the common crop foragers in WWNSF in their ranking order were grivet monkeys (47.6%), bushbuck (37.8%), gelada (5.6%), porcupine (4.2%), rabbit (2.8%) and duiker (2.1%), where grivet monkeys were the most intensive. Accordingly, the majority of respondents from both genders (male = 67.1%; female = 74.1%) did not support grivet monkey conservation indicating that they have negative attitudes toward these monkeys. More male (32.9%) respondents were interested in the conservation of grivet monkeys as compared with females (20.7%). Pearson’s Chi-Square test showed that there was a significant difference between genders regarding their interest in grivet monkey conservation (χ2 = 6.49, df = 2, *P* = 0.04) (Table 2). There was no statistical difference in respondents’ perceptions of grivet monkey conservation based on their marital status, education status, and family size.

Respondents’ village and their cropland distance from the forest had a significant impact on their support for grivet monkey conservation. The majority of respondents (77.8%) that had farmland 401 m away from the forest supported the importance of grivet monkey conservation while those nearer to the forest argued against the issue. There was a significant difference in respondents’ perceptions towards grivet monkey conservation based on the distance of farmland from the forest (χ2 = 12.7, df = 4, *P* = 0.013) (Table 2). Respondents who argued against grivet monkey conservation claimed several problems like damage to field crops, gardens, theft of backyard resources, children’s absenteeism from schooling, and extra workload of crop guarding which interrupt other socioeconomic activities. On the other hand, respondents who supported grivet monkey conservation stated that the monkeys are a source of happiness, tourist attraction, and heritage to the country and the world for the generations to come.

**Table 2:** Community perceptions towards grivet monkey conservation around WWNSF

		Is conserving grivet monkey important?					
Variables	Categories	Important (%)	Not important (%)	I do not know (%)	χ2	df	*P* value
Sex	male	32.9	67.1	0.0	6.49	2	0.04
	female	20.7	74.1	5.2			
Age	18–30	24.4	75.6	0.0	13.23	6	0.04
	31–43	25.0	75.0	0.0			
	44–56	35.5	54.8	9.7			
	> 56	30.4	69.6	0.0			
Marital status	married	25.9	71.6	2.6	1.91	2	0.39
	single	37.0	63.0	0.0			
Educational status	Uneducated	21.7	73.9	4.3	3.53	6	0.74
	informal education	25.0	75.0	0.0			
	primary	32.0	66.0	2.0			
	secondary	31.8	63.6	4.5			
Distance from forest (m)	< 200	24.5	72.6	2.8	12.7	4	0.013
	201–400	25	75	0.0			
	> 401	77.8	22.2	0.0			
Family size	1–3	36.1	63.9	0.0	7	4	0.13
	4–6	20.0	76.0	4.0			
	> 7	37.5	62.5	0.0			
Village	Chachahudad	41.2	58.8	0.0	22	10	0.013
	Giderach-Lankuso	13.6	86.4	0.0			
	Ayer	8.0	92.0	0.0			
	Silasie Gedam	38.7	58.1	3.2			
	Mebreka`mba	42.1	47.4	10.5			
	Gifte	27.6	72.4	0.0			

**Fig. 1:**
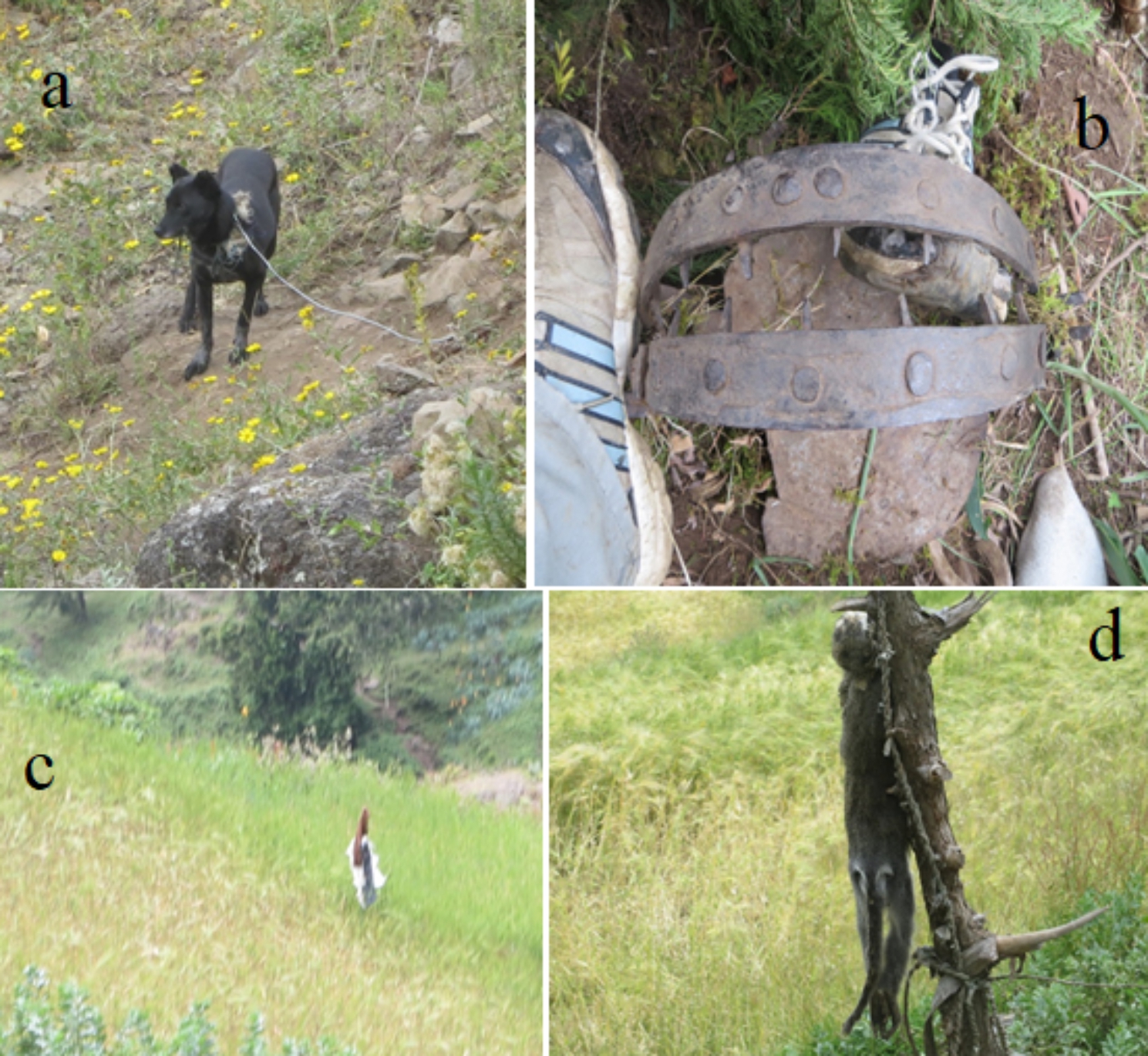
Grivet monkey crop damage protection techniques around Wof Washa Natural State Forest (WWNSF): (a) Dog tied at the border of farmland to alarm arrival of grivet monkey; (b) Trapping tool fixed for wildlife wedged up a person foot; (c) Scarecrow at wheat farmland (d) Killed grivet monkey hanged on a tree at the border of farmland

Among the various techniques (guarding by dog, guarding and scare away by humans, scarecrow, and killing by trap) (Fig. 1a-d) used to prevent crop damage by grivet monkeys, 51.3% ± SD 15.2 (n = 74) of respondents used humans to scare away grivets (Table 3). Although, killing was the least (7.2 ± SD 2.5) used technique, it has been used for two reasons: (1) to reduce the number of monkeys and (2) to chase away troops of grivet monkeys and discourage further crop foraging by using the dead animals as a scarecrow (Fig. 1d). There was no significant difference in the techniques used by villagers to alleviate crop damage by grivet monkeys (χ2 = 14.73, df = 15, *P* = 0.47) (Table 3). Guarding using dogs was the second preferred and used technique. Farmers either moved with dogs or tied dogs at the periphery of farmland to function as an alarm for farmers (Fig. 1a). Human guarding and scaring includes: watching, chasing, shouting, and using slingshots to scare-grivet monkeys back into the forest. Females and children are the primary family members to guard crop fields against crop foraging.

**Table 3:** Techniques used by respondents to prevent crop damage by grivet monkey around WWNSF

	Method of crop protection			
Village	Guarding by dog (%)	Guarding & scare away by humans (%)	Scarecrow (%)	Killing by trap (%)
Chachahudad	17.6	58.8	17.6	5.9
Giderach-Lankuso	31.8	36.4	22.7	9.1
Ayer	44.0	28.0	20.0	8.0
Silasie Gedam	22.6	64.5	6.5	6.5
Mebrekamba	15.8	57.9	15.8	10.5
Gifte	24.1	62.1	10.3	3.4
Mean	26.0	51.3	15.5	7.2
Standard Deviation (SD)	10.5	15.2	6.1	2.5

**Fig. 2:**
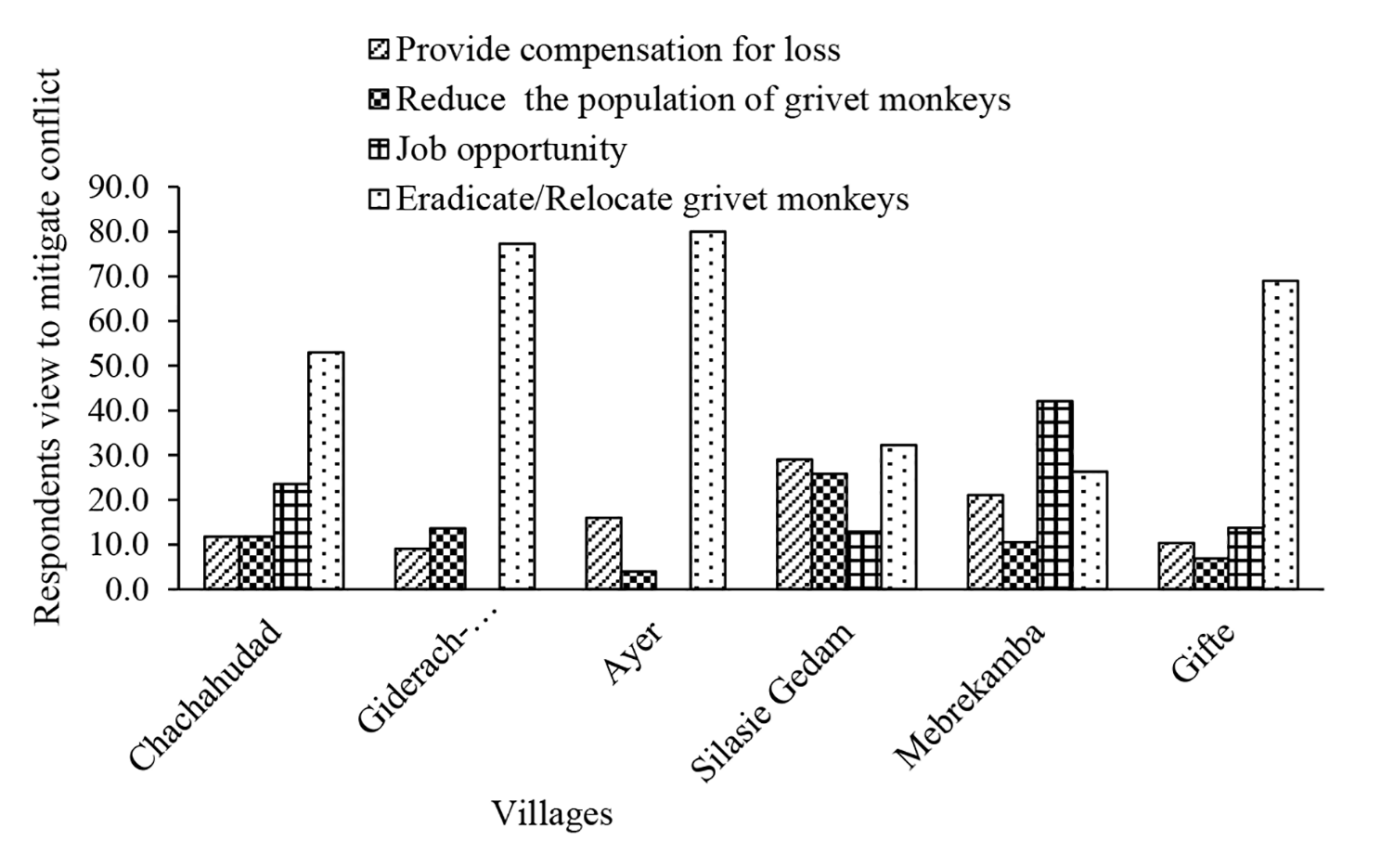
Respondents’ opinion on the options to be taken by the government and other stakeholders

The respondents expect stakeholders, including government bodies, to design alternative crop damage prevention methods and compensation of damage to reduce HGMC. The households revealed that they would be delighted if the government took measures like compensation strategies, reduction of the number of grivet monkeys, and relocation options. The majority of respondents (56.3% ± SD 23) claimed eradication/relocation of grivet monkeys followed by financial compensation (16.2 ± SD 7.7) including an exemption from farmland taxation (Fig. 2). There was a significant difference in respondents’ views on the mitigation measures to be taken by the government (χ2 = 40.01, df = 15, *P* = 0.000).

Respondents claimed that habitat degradation, the proximity of cropland to forest, preferences of grivet monkeys to crops, and the depletion of grivet monkeys’ food plants in the area were the main causes of crop damage. On average 42.5 ± SD 8.68 of respondents in all villages elucidated that the causes of crop damage by grivet monkeys were triggered by habitat degradation (Table 4). The proximity of cropland to the forest was rated as the second reason for crop damage by grivet monkeys and associated conflict. There was no significant difference in the respondents’ views on the causes of crop damage by grivet monkeys among villages (χ2 = 6, df = 15, *P* = 0.98).

**Table 4:** Respondents’ perception on the causes of crop damage by grivet monkeys around WWNSF

Village	Habitat degradation	Preferences to crop	Proximity of farmland to forest	Food plants degradation
Chachahudad	58.8	5.9	11.8	23.5
Giderach-Lankuso	36.4	4.5	31.8	27.3
Ayer	44.0	12.0	24.0	20.0
Silasie Gedam	35.5	6.5	35.5	22.6
Mebrekamba	42.1	5.3	31.6	21.1
Gifte	37.9	10.3	31.0	20.7
Mean	42.5	7.4	27.6	22.5
SD	8.68	3.02	8.62	2.66

**Fig. 3:**
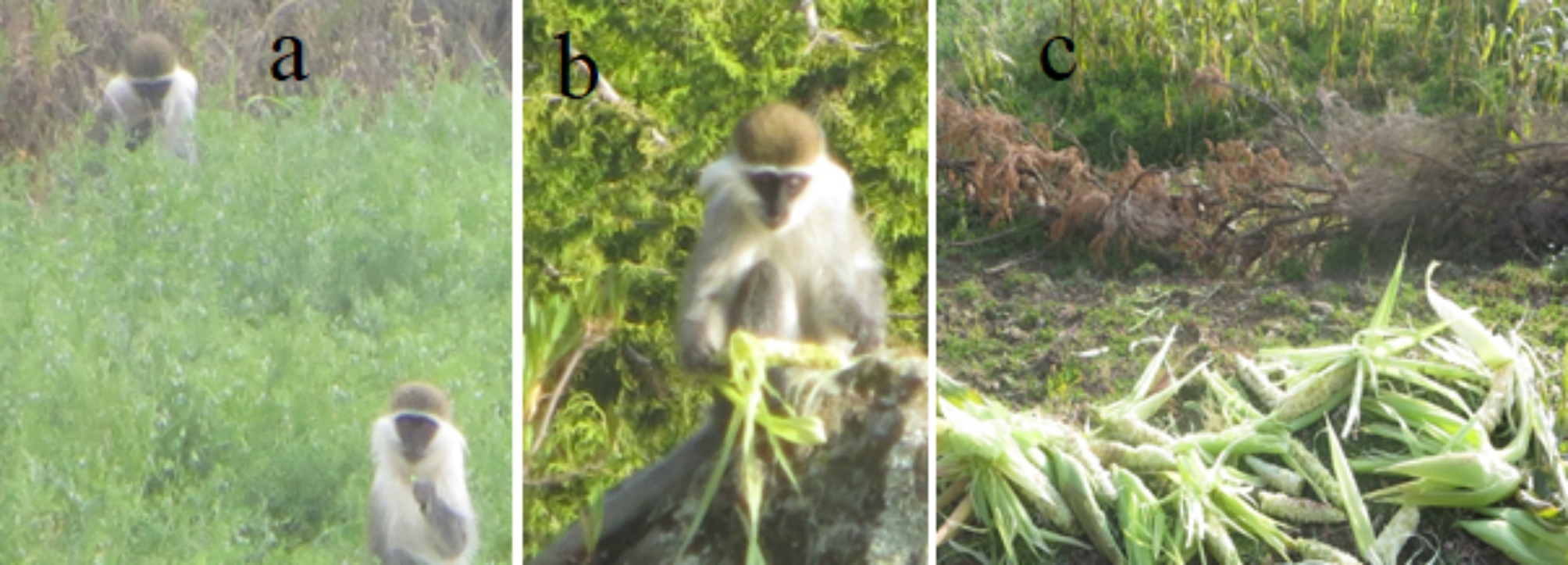
Events of crop foraging by Grivet monkeys around Wof Washa Natural State Forest (WWNSF): (a) Grivets feeding on lentil farm; (b) Grivet monkey feeding on snatched maize; (c) Leftover of consumed maize gathered from maize farm

Grivet monkeys have been persecuted by humans as a consequence of crop foraging. Respondents chase, shoot, and trapped grivet monkeys as retaliation for their crop loss (Fig. 3a-c). Residents around WWNF usually cut trees such as *Hagenia abyssinica, Prunus Africana, Olea europaea cuspidata, Olinia rochetiana, Ficus sur, Dombeya torrid, Myrica salcifolia, Allophylus abyssinicus, Ekebergia capensis, Podocarpus falcatus, Juniperus procera, Maesa*.

*lanceolata, Ilex mitis, Celtis Africana*, and others for purposes like house construction, firewood, timbering, animal fodder, fencing and household and farming utensils (Table 5), most of which were used as food sources for grivet monkeys. Such overlapping of human goals with the needs of grivet monkeys’ further escalates the conflict and endangers grivet monkey conservation in the study area.

**Table 5:** Tree species logged by local communities around WWNF for different purpose

		Forest product utilization			
Local name	tree species	timbering for house & utensil construction	fuel wood	animal fodder	Fencing
Zigba	*Podocarpus falcatus*	√	√		
Tsid	*Juniperus procera*	√			√
Zingerowonber	*Polyscias fulva*	√			
Misargenfo	*Ilex mitis*			√	
Weira	*Olea europaea*	√	√	√	
Kelewa	*Maesa lanceolata*		√		√
Weyel	*Pittasporum viridiflorum*	√	√	√	√
Kosso	*Hygenia abyssinica*	√		√	
Tifie	*Olinia rochetiana*	√	√	√	√
Ameja	*Hypericum revolutum*		√	√	
Shola	*Ficus Sur*				
Wulkifa	*Dombeya torrida*			√	
Azamir	*Bersama abyssinica*		√		
Kewot	*Celtis Africana*		√		
Lanquso	*Urera hypselodendron*				
totakula	*Galiniera saxifraga*	√	√	√	
Embus	*Allophylus abyssinica*		√		
Kechemo	*Myrsine africana*			√	√

## Discussion

In recent decades, human population growth and the related expansion of agricultural and industrial activities have intensified HWCs [18, 19]. In developing countries like Ethiopia, the livelihoods of most people living in rural areas are dependent upon livestock holdings and agriculture which intensify HWC [3]. Recently, HWC has undoubtedly ranked among the main threats to wildlife conservation in Africa [11]. This study also revealed that the communities around WWNSF experienced intensive conflict with grivet monkeys over crops. The livelihoods of all communities in the study area were subsistence farming where they reared livestock and cultivated different crops on small land sizes [16]. The households in the study area possess small plots of farmlands where they cultivated their subsistence production. Another similar result reported that most of the respondents produce a limited number of crops on plots of land [18].

The present study revealed that human**-**grivet monkeys’ conflict is a customary interaction within the WWNSF boundary. In spite of several factors, crop foraging is the root cause of increasing human primate conflict during the months from sowing crops to harvesting. However, the forerunner cause of human-wildlife conflict in the study area is habitat encroachment accompanied by the fragmentation of wildlife habitats including primates (grivet monkeys). Humans have two basic impacts on wildlife (grivet monkeys) (i) encroachment to wildlife habitat for farmland expansion (ii) degrading the quality of habitat by logging and livestock grazing in the forest. Eventually, the increased pressure on animals to find food leads them to visit and forage on croplands resulting in Human-Wildlife conflicts. The attitudes of respondents to the conservation of grivet monkeys were negative and differences were observed with reference to gender, distance from the forest, and village/localities of cropland relative to the forest. The difference in the perception of gender towards grivet monkey conservation is possibly due to the more involvement of females in guarding crops against grivet monkeys which causes them to interact with the animals [2]. Respondents having cropland closer to the forest area had negative attitudes towards grivet monkey conservation due to economic impact, replicating the trend of crop damage reported from earlier studies [2, 13, 16, 20, 21]. Respondents are used to chasing out the monkeys from the periphery of the forest facing the cropland. This is opposed to the thought that resolving HWC lies in avoiding wildlife attractions to human habitation instead of getting rid of monkeys, which indeed requires a change in human habits and invincible ways of thinking [22].

Households around WWNSF prevent crop damage against grivet monkeys by guarding their cropland using scarecrows including carcasses of killed grivet monkeys [21], dogs, children and females vigilance, and even trapping animals. Out of the various techniques used in discouraging grivet monkeys from crop foraging, guarding by humans was a highly preferred one [2, 12]. Moreover, studies revealed that guarding is the most effective and commonly used method to prevent crop damage from crop foragers [2, 18, 20, 21, 23]. Killing was the least used method in this study area where the action is forbidden by the law of the country to protect the wildlife from mass persecutions. Moreover, scarecrows were ineffective at deterring grivet monkeys from crop damage as the animals habituated to the symbol and become non-responsive after a certain timeframe. A similar study has reported that the effect of scarecrows in preventing crop damage by wildlife is temporary [12].

Crop-foraging by wildlife like grivet monkeys is perceived as a significant problem causing a serious hazard to the food security and livelihoods of smallholder farmers’ households [20], which leads households to develop negative attitudes and resentful attacks on primates. Understanding the attitude of local communities towards wildlife is crucial to attaining long-term conservation success [24]. The questionnaire survey revealed that the anticipated perceptions of respondents to tackle HGMC in WWNSF were eradication and relocation of grivet monkeys followed by financial compensation. Moreover, they would be thrilled if government bodies intervene to reduce the population size of the animals by killing them. Similarly, other studies reported that relocation and killing of problematic wildlife were the actions that households recommend to combat HWC that they experienced in Ngangao forest, Kenya [20]. Developing mitigation measures, accurate and reasonable verification of damages, and compensation schemes to economic impacts are the priority areas implemented to reduce the resource damages posed by wildlife [25]. Compensation for damage is strong enough to increase the productivity of farmers and decrease the perceived HWC although the procedure of compensation is excruciating and difficult because of the mutual mistrust among farmers and government authorities [3, 25–27]. Whatever the cases, implementing realistic compensation has positive effects and provides immediate relief and reasonable conciliation for farmers struggling with direct and chronic losses due to wildlife [3, 26, 27].

Respondents opined that the main cause of crop damage by grivet monkeys in WWNSF was triggered by habitat degradation. Local communities encroach upon the forest to collect wood for sale, for fuel, animal fodder, and household and agricultural utensils. This finding is similar to studies conducted on red-tailed monkeys in Uganda [28] and on grivet monkeys in Batiero Church forest, Ethiopia [16], inferring that the conversion of forests for agricultural farmlands and other purposes resulted in nonhuman primate crop foraging. Respondents in our study did not consider food preference as a reason for grivet monkey crop foraging. In line with this finding, [10] in Lake Nabugabo, Uganda, reported that nutritionally anthropogenic and wild food are similar. However, [16] in Batiero Church forest, Ethiopia, reported that human-primate conflicts were intensified during crop maturity and harvesting stages when grivet monkeys had a preference to feed on the crops which differed from this result. This might be due to forest patchiness, degradation and lower quality of the habitat in Batiero Church where reduction in the availability of natural food sources has led animals to seek alternative food sources [23]. Accordingly, human expansion into natural habitats worldwide has been the root cause of the intense conflict between wildlife and humans where natural food sources of wildlife are replaced by anthropogenic ones [22].

## Conclusion

The finding of this study revealed that there was a strong conflict between human and grivet monkeys in and around WWNSF. This study, therefore, provides baseline data on crop damage for developing a feasible wildlife management plan to enable friendly long-term coexistence of local communities with grivet monkeys and other wildlife in and around WWNSF. Because crop-foraging by wildlife including grivet monkeys is the main cause that leads local communities to develop negative attitudes and engaged in retaliatory killing of wildlife that could lead to local extermination. Hence, grivet monkeys have been impacted by human persecution as a consequence of crop foraging. Forest conversion into agricultural lands and degradation due to cutting of trees for sale and firewood represents the most detrimental human activity that triggers HWC. Mitigation measures of HGMC need to focus on techniques that would not result in local extermination of grivet monkey populations rather simply deter these animals in such a way that they stay in the forests, their natural home. Farmers also need to be encouraged to shift their crop productions to those which are unpalatable to grivet monkeys. More education can be launched to create awareness among local communities on the importance of wildlife and create job opportunities to reduce unemployment to mitigate the pressure of local people on wildlife and the forest.

## Methods

### Description of the study area

**Fig. 4:**
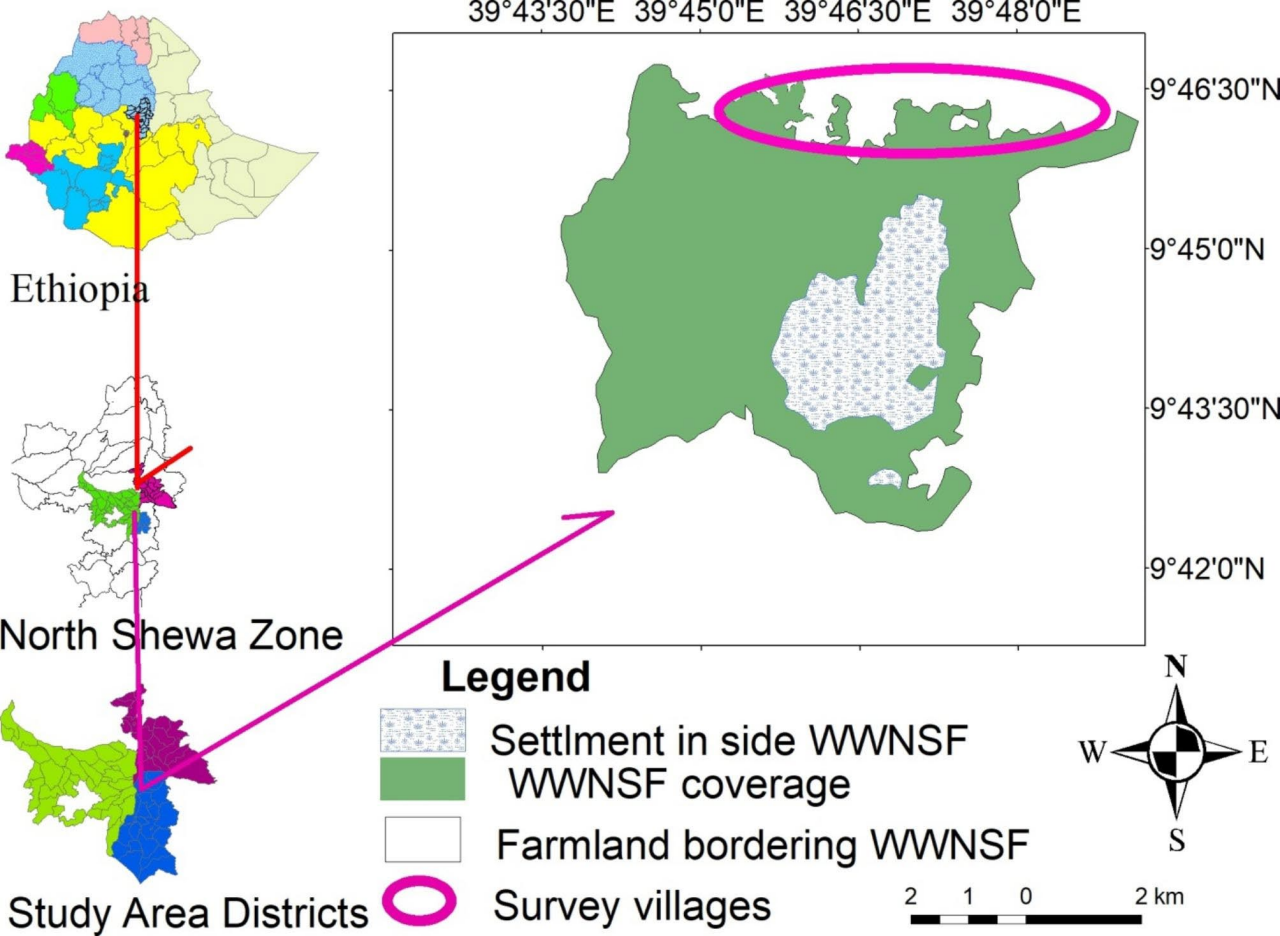
Map of Wof Washa Natural State Forest (WWNSF)

The study on HGMC and community perception to crop loss by grivet monkeys (*Cercopithecus aethiopes aethiops)* was conducted in and around Wof-Washa Natural State Forest (WWNSF), which is located in North Shoa Zonal administration, Amhara National Regional State, Northwestern highlands of Ethiopia. The study area extends approximately between 9º42′- 9º47′ N latitudes and between 39 º 43′- 39 º 49′E longitudes (Fig. 4). Topographically, the forest is situated along the altitudinal gradient between 1,650 m asl near Gift Michael where it merges into *Acacia* scrubland on the valley floor to 3,700 m asl at the top of the Rift Valley escarpment near Kundi on the plateau ridge.

The long history of settlement and cultivation coupled with deforestation and cattle grazing have led to intense pressure on the land, decreased soil quality, soil erosion, and deforestation. Beyond the hillsides are mostly very steep and hard to cultivate and plot sizes are commonly very small with an average land holding ~ 1.5 ha in Amahara Region [29].

The main characteristic plant species at the higher altitudes are *Hagenia abyssinica*, *Olea europaea cuspidata* and *Juniperus procera*. *Podocarpus falcatus, Allophylus abyssinicus, Haleria lucida, Euphorbia abyssinica, Polyscias fulva* and *Olinia rochetiana* at the middle. Above 3,000 m *Erica arborea*, *Hypericum revolutum* and giant *Lobelia* spp. are the most dominant species with few *Hagenia abyssinica* and *Pittosporium viridiflorum* below inaccessible cliffy and steep slope areas. There are over 394 species of plants, of which, 46 species (12%) are endemic to Ethiopia while 7 (2%) are nearly endemic [30, 31].

### Data collection

In order to evaluate the perceptions of farmers on crop damage by grivet monkeys, the researcher surveyed villagers living around WWNSF from August 2017 to June 2018. Questionnaire surveys were administered to 143 households in six grivet monkey localities near WWNSF (Ayer, Chachahudad, Giderach-Lanquso, Silasie Gedam, Mebreqamba and Gifte). These households were randomly selected by following a pattern of skipping one household, and the second household was interviewed. The survey questionnaires were administered to farmers within their farming area or residence by the researcher and field assistants [32]. Questionnaires included both open and close-ended questions to gain information about communities’ perceptions on HGMC, their socioeconomic situation, measures they took to mitigate losses, and attitudes to grivet conservation. One person at least 18 years of age was interviewed to represent a household and the average interview session per sampled household was 25–35 min.

### Data analysis

Data were analyzed using Statistical Package for Social Sciences (SPSS) software version 25. Descriptive statics and Pearson Chi-Square test were used to analyze the data. Chi-square test was used to determine the significant differences among villages with regard to perceptions to crop damage by grivet monkeys, techniques used in protecting against crop damage, and attitudes towards conservation of grivet monkeys. All statistical tests were two-tailed with 95% confidence intervals and a level of rejection set at P = 0.05.

## Electronic supplementary material

Below is the link to the electronic supplementary material.

Supplementary Material 1

## Data Availability

All data generated or analyzed during this study are included in this published article.

## Abbreviations

GPS: Global Positioning System.
HGMC: Human-Grivet Monkey Conflict.
HWC: Human-Wildlife Conflict.
SD: Standard Deviation.
SPSS: Statistical Package for Social Sciences.
WWNSF: Wof-Washa Natural State Forest.
