# Supplementary material for: Human-wildlife conflict and community perceptions towards wildlife conservation in and around Wof-Washa Natural State Forest, Ethiopia
Source: BMC Zool. 2022 Sep 27;7:53. doi: 10.1186/s40850-022-00154-5 (PMC10127036; doi:10.1186/s40850-022-00154-5)
Supplement: Supplementary file 1 — Supplementary Material 1 [file 40850_2022_154_MOESM1_ESM.docx]

Annex 1. Raw data of figure 2 (Respondents view on the options to be taken by the government and other stakeholders)

|  | Respondents view to mitigate conflict | | | |
| --- | --- | --- | --- | --- |
| Village | Provide compensation for loss | Reduce the population of grivet monkeys | Job opportunity | Eradicate/Relocate grivet monkeys |
| Chachahudad | 11.8 | 11.8 | 23.5 | 52.9 |
| Giderach-Lankuso | 9.1 | 13.6 | 0.0 | 77.3 |
| Ayer | 16.0 | 4.0 | 0.0 | 80.0 |
| Silasie Gedam | 29.0 | 25.8 | 12.9 | 32.3 |
| Mebrekamba | 21.1 | 10.5 | 42.1 | 26.3 |
| Gifte | 10.3 | 6.9 | 13.8 | 69.0 |
| **Average** | 16.2 | 12.1 | 15.4 | 56.3 |
| **SD** | 7.7 | 7.6 | 15.9 | 23.0 |
